# Supplementary material for: New Acylcarnitine Ratio as a Reliable Indicator of Long-Chain 3-Hydroxyacyl-CoA Dehydrogenase Deficiency
Source: Int J Neonatal Screen. 2023 Aug 25;9(3):48. doi: 10.3390/ijns9030048 (PMC10531771; doi:10.3390/ijns9030048)
Supplement: Supplementary file 1 [file IJNS-09-00048-s001.zip › IJNS-2558440-supplementary.pdf]

Supplementary Material

Table S1. Acylcarnitine concentrations (μmol/L) and genetic data of the study cohort

Patient	Sex	Age at blood collection (rationale)	C0	C16	C16:1OH	C16OH	C18:1OH	C18OH	"HADHA ratio"	C16OH/C16	Genotype
1	M	4 days (initial clinical symptoms)	35,3	2,64	0,156	1,09	0,547	1,03	0,076	0,413	c.1528G>C, (p.Glu510Gln) homo
2	F	4 days (initial clinical symptoms)	58,1	2,11	0,086	0,863	0,666	1,05	0,044	0,409	c.1528G>C, (p.Glu510Gln) homo
3	M	4 days (initial clinical symptoms)	43,6	3,82	0,554	1,97	2,03	2,29	0,144	0,516	c.1528G>C, (p.Glu510Gln) homo
4	F	7 days (initial clinical symptoms)	43,5	3,17	0,44	1,36	0,776	0,899	0,070	0,429	c.1528G>C, (p.Glu510Gln)/c.2112_2114dup
5	M	7 days (initial clinical symptoms)	20,1	2,24	0,142	0,745	0,791	0,452	0,099	0,333	c.1528G>C, (p.Glu510Gln) homo
6	M	1 month (asymptomatic)	10,581	1,104	0,089	0,209	0,117	0,175	0,047	0,189	c.1528G>C, (p.Glu510Gln)/ c.67G>C (p.Gly23Arg)

7	F	20 days (asymptomatic)	24,642	4,079	0,331	1,749	1,275	1,685	0,191	0,429	c.1528G>C, (p.Glu510Gln) homo
8	M	1 month (initial clinical symptoms)	22,124	0,954	0,131	0,506	0,612	0,742	0,084	0,530	c.1528G>C, (p.Glu510Gln) homo
9	M	2 months (metabolic decompensation)	53,975	1,929	0,366	1,31	1,366	1,597	0,079	0,679	c.1528G>C, (p.Glu510Gln) homo
10	F	10 days (neonatal screening, asymptomatic)	12,696	0,963	0,108	0,398	0,246	0,25	0,07	0,413	c.1528G>C, (p.Glu510Gln) homo
11	M	3 months (metabolic crisis)	3,7	1,18	0,169	0	0,202	0,322	0,142	0,000	c.1528G>C, (p.Glu510Gln)/ c.1750_1772del (p.Ala584TrpfsTerm40)
12	M	3 months (metabolic crisis)	11,427	1,082	0,243	0,752	0,707	0,771	0,195	0,695	c.1528G>C, (p.Glu510Gln) homo
13	F	4 months (metabolic crisis)	5,306	1,665	0,168	0,59	0,677	0,37	0,308	0,354	c.1528G>C, (p.Glu510Gln) homo
14	M	4 months (metabolic crisis)	7,32	0,924	0,179	0,447	0,474	0,498	0,194	0,484	c.1528G>C, (p.Glu510Gln)/ c.871C>T (p.Arg291Term)

15	F	5 months (metabolic crisis)	26,01	2,397	0,563	1,5	2,435	2,17	0,235	0,626	c.1528G>C, (p.Glu510Gln)/ c.1493A>G
16	M	3 months (clinical symptoms)	11,837	1,681	0,285	0,776	1,139	0,715	0,222	0,462	c.1528G>C, (p.Glu510Gln) homo
17	M	5 months (metabolic crisis, L-carnitine theraphy)	384,663	11,849	3,49	16,374	13,29	11,017	0,106	1,382	c.1528G>C, (p.Glu510Gln) homo
18	M	5 months (clinical symptoms)	9,073	0,947	0,211	0,692	1,114	0,623	0,268	0,731	c.1528G>C, (p.Glu510Gln) homo
19	M	6 months (clinical symptoms, metabolic decompensation)	3,13	0,456	0	0,228	0,336	0,432	0,318	0,500	c.1528G>C, (p.Glu510Gln) homo
20	F	6 months (metabolic crisis)	17,976	2,676	0,5	0,881	1,773	1,074	0,207	0,329	c.1528G>C, (p.Glu510Gln) homo
21	M	6 months (metabolic crisis)	5,274	1,504	0,181	0,494	0,637	0,546	0,318	0,328	c.1528G>C, (p.Glu510Gln) homo
22	M	6 months (clinical symptoms, metabolic	3,581	0,616	0,086	0,217	0,345	0,239	0,224	0,352	c.1528G>C, (p.Glu510Gln) homo

		decompensation)									
23	M	6 months (clinical symptoms)	18,301	1,923	0,146	0,495	0,466	0,508	0,08	0,257	c.1528G>C, (p.Glu510Gln) homo
24	F	6 months (metabolic crisis)	3,323	0,964	0,175	0,453	0,514	0,456	0,428	0,470	c.1528G>C, (p.Glu510Gln) homo
25	M	6 months (clinical symptoms)	35,8	4,127	0,446	1,669	0,889	0,577	0,088	0,404	c.2026C>T, (p.Arg676Cys)/ c.2134_2138dupCCTTG (p.Gly715fs)
26	M	6 months (clinical symptoms)	16,271	2,623	0,298	0,721	0,655	0,491	0,115	0,275	c.1528G>C, (p.Glu510Gln) homo
27	M	7 months (metabolic crisis)	2,275	0,605	0,354	0,428	0,432	0,391	0,55	0,707	c.1528G>C, (p.Glu510Gln)/ -**
28	M	6 months (metabolic crisis)	3,933	1,243	0,226	0,798	1,157	0,342	0,584	0,642	c.1528G>C, (p.Glu510Gln) homo
29	M	7 months (clinical symptoms)	11,114	1,695	0,39	1,018	1,559	0,64	0,289	0,601	c.1528G>C, (p.Glu510Gln) homo
30	M	7 months (clinical	5,765	1,127	0,172	0,345	0,706	0,7	0,304	0,306	c.1528G>C, (p.Glu510Gln) homo

		symptoms)									
31	F	7 months (clinical symptoms)	3,934	1,186	0,181	0,498	0,757	0,647	0,483	0,420	c.1528G>C, (p.Glu510Gln) homo
32	F	7 months (metabolic crisis)	6,364	1,507	0,265	0,933	0,898	0,888	0,427	0,619	c.1528G>C, (p.Glu510Gln) homo
33	M	4 days (metabolic crisis)	43,105	5,077	0,251	0,76	0,369	0,406	0,036	0,150	c.1528G>C, (p.Glu510Gln)/ -**
34	F	8 months (clinical symptoms)	8,94	0,704	0	0,217	0,289	0,505	0,113	0,308	c.1528G>C, (p.Glu510Gln) homo
35	M	8 months (clinical symptoms)	5,7	0,469	0	0,073	0,12	0,12	0,055	0,167	c.1528G>C, (p.Glu510Gln) homo
36	F	8 months (clinical symptoms)	5,466	0,796	0,255	0,47	1,027	0,696	0,401	0,590	c.1528G>C, (p.Glu510Gln) homo
37	M	8 months (clinical symptoms)	7,44	1,73	0,141	0,424	0,661	0,418	0,202	0,245	c.1528G>C, (p.Glu510Gln) homo
38	M	8 months (clinical symptoms)	14,688	0,557	0,111	0,241	0,219	0,154	0,042	0,433	c.1528G>C, (p.Glu510Gln)/ c.67G>C (p.Gly23Arg)

39	F	8 months (clinical symptoms)	2,69	0,308	0,078	0,157	0,342	0,184	0,254	0,510	c.1528G>C, (p.Glu510Gln) homo
40	F	8 months (clinical symptoms)	4,511	0,789	0,108	0,203	0,501	0,252	0,212	0,257	c.1528G>C, (p.Glu510Gln) homo
41	F	9 months (clinical symptoms)	2,875	0,292	0,033	0,084	0,073	0,044	0,07	0,288	c.1528G>C, (p.Glu510Gln) homo
42	M	9 months (clinical symptoms)	41,42	2,3	0,478	1,62	2,093	2,006	0,138	0,704	c.1528G>C, (p.Glu510Gln)/ -**
43	M	9 months (clinical symptoms)	2,463	0,357	0,094	0,182	0,393	0,178	0,305	0,510	c.1528G>C, (p.Glu510Gln) homo
44	M	9 months (clinical symptoms)	6,146	0,387	0,07	0,239	0,405	0,408	0,171	0,618	c.1528G>C, (p.Glu510Gln) homo
45	M	7 months (clinical symptoms)	11,611	0,369	0	0,114	0,354	0,236	0,061	0,309	c.1528G>C, (p.Glu510Gln) homo
46	F	7 months (clinical symptoms)	35,2	1,58	0	0,542	0,643	0,778	0,056	0,343	c.1528G>C, (p.Glu510Gln) homo

47	F	10 months (clinical symptoms)	45,7	5,08	0,425	1,91	1,06	1,73	0,103	0,376	c.1528G>C, (p.Glu510Gln)/ -**
48	M	5 days (no symptoms)	23,742	3,897	0,101	0,402	0,224	0,505	0,048	0,511	c.1528G>C, (p.Glu510Gln) homo
49	M	10 months (metabolic crisis)	3,439	0,438	0,08	0,145	0,278	0,216	0,186	0,331	c.1528G>C, (p.Glu510Gln) homo
50	M	10 months (clinical symptoms)	14,064	1,506	0,414	1,205	3,132	1,282	0,400	0,800	c.1528G>C, (p.Glu510Gln) homo
51	F	10 months (clinical symptoms)	8,103	0,718	0,093	0,204	0,392	0,239	0,103	0,284	c.1528G>C, (p.Glu510Gln) homo
52	M	85 months (clinical symptoms)	7,283	0,539	0,064	0,09	0,08	0,135	0,042	0,167	c.1528G>C, (p.Glu510Gln) homo
53	M	166 months (metabolic decompensation, rhabdomyolysis)	32,9	0,882	0,060	0,261	0,357	0,524	0,035	0,296	c.1528G>C, (p.Glu510Gln)/ c.482C>A (p.Ala161Glu)
54	M	318 months (clinical symptoms)	5,75	1,22	0,279	0,768	0,511	0,316	0,277	0,630	c.1528G>C, (p.Glu510Gln) homo

RI	8 – 90	0,11 - 6,3	< 0.22	< 0.18	< 0.16	< 0.15	< 0.027	<0.1*	

Symbols and abbreviations are as follows: “HADHA ratio” = (C16OH + C18OH + C18:1OH)/C0; RI = reference intervals; ** = unidentified mutation due to a refusal of a patient’s parents/ legal guardian to proceed the research due to the patient’s death. * = values taken from McHugh and coauthors [26]. Control group genotyping was not performed.

Table S2. Clinical data of the study cohort

Patient	Sex	Age at diagnosis (months)	Age at death (months)	Family history	First symptoms	Phenotype (according to the age of onset and disease severity)	Main clinical symptoms
1	M	Retrospective diagnosis	8	Positive (two affected siblings - patients 1 and 2)	Within the first 24 h of life	Severe	Hypoglycemia, cardiomyopathy, hepatic impairment, sudden infant death syndrome (SIDS)
2	F	Retrospective diagnosis	7	Positive (two affected siblings - patients 1 and 2)	Within the first 24 h of life	Severe	Hypoglycemia, hypotonia, cramps, anemia
3	M	1	1	Unknown	Within the first 24 h of life	Severe	Hypoglycemia, hypotonia, cramps
4	F	1	1	Negative	Within the first 24 h of life	Severe	Hypoglycemia, hypotonia, cramps
5	M	1	Alive	Positive (the sibling died within the first year of life)	Within the first months of life	Intermediate (precipitated by infection)	Hypoglycemia, hypotonia, cramps, symptom improvement after therapy

6	M	8	Alive	Negative	At the age of 7 months - hypotonia, hepatic cytolysis, disorders of consciousness	Intermediate (precipitated by infection)	Hypoglycemia, hepatic impairment (hepatomegaly, hepatic cytolysis), hypotonia
7	F	7	Unknown	Negative	At the age of 7 months - hypoglycemia	Intermediate (precipitated by infection)	Hypoglycemia, cramps, hypotonia, hepatomegaly, hepatic cytolysis
8	M	1	6	Negative	Within the first months of life - hypoglycemia, hepatic cytolysis	Severe	Hypoglycemia, cramps, hypotonia, hepatomegaly, hepatic cytolysis, cardiomyopathy
9	M	2	Alive	Negative	Within the first 24 h of life - respiratory disorders, hypotonia	Severe	Hypoglycemia, dilated cardiomyopathy, anemia
10	F	0 (10 days)	Alive	Negative	Preclinical stage (diagnosed by neonatal screening)	Asymptomatic	No symptoms. The patient is receiving therapy
11	M	2	3	Unknown	Within the first months of life - hepatic impairment, hepatic cytolysis, hypoglycemia	Severe	Hypoglycemia, hepatic cytolysis, hypocoagulation
12	M	3	3	Unknown	Unknown	Severe	Hypoglycemia, hepatic impairment (hepatomegaly, hepatic cytolysis), hypotonia
13	F	4	Unknown	Positive (the sibling died of hepatic impairment at the age of 7 months)	Since birth - mild regurgitation. At the age of 3 months - vomiting, hypoglycemia	Severe	Hypoglycemia, hypotonia, cardiomyopathy, hepatic cytolysis, anemia, hypocoagulation

14	M	4	Alive	Positive (two siblings, twins). The boy and girl died in 8 and 14 h after birth, respectively	At the age of 3 months - cramps, prolonged apnea	Severe	Hypotonia, hepatomegaly, hypoglycemia, cardiomyopathy, hepatic cytolysis
15	F	5	Unknown	Negative	At the age of 5 months - lethargy, vomiting	Intermediate (precipitated by infection)	Hypotonia, hepatomegaly, hypoglycemia, cardiomyopathy, hepatic cytolysis
16	M	5	Alive	Negative	At the age of 5 months - hepatomegaly	Intermediate (precipitated by infection)	Hepatomegaly, hepatic cytolysis, anemia
17	M	5	5	Positive (the sibling died at the age of 6 months)	At the age of 4 days - enterocolitis with intestinal perforation	Intermediate (precipitated by infection)	Increased lactate level, cardiomyopathy, myocardial hypertrophy, hypoglycemia, hypocoagulation, hepatic cytolysis
18	M	Retrospective diagnosis	6	Positive (sibling)	Within the first days of life - hypoglycemia, hepatic cytolysis	Intermediate (precipitated by infection)	Hypoglycemia, hepatic cytolysis, cardiomyopathy
19	M	6	Unknown	Unknown	Hypoglycemia, hepatic cytolysis	Intermediate (precipitated by infection)	Hypoglycemia, hypotonia, cardiomyopathy, hepatic cytolysis
20	F	6	7	Negative	At the age of 6 months - hypoglycemia, hepatic impairment	Intermediate (precipitated by infection)	Hypoglycemia, disorders of consciousness, hypotonia, cardiomyopathy, lactic acidosis, hepatic cytolysis, hypocoagulation
21	M	6	6	Unknown	At the age of 6 months - hypoglycemia, hepatic cytolysis	Intermediate (precipitated by infection)	Hepatic impairment, hepatic cytolysis

22	M	6	Alive	Negative	Within the first days of life - jaundice. At the age of 6 months - developmental delay, hypotonia	Intermediate (precipitated by infection)	Hypoglycemia, epilepsy, motor impairment
23	M	6	Unknown	Unknown	Unknown	Intermediate (precipitated by infection)	hepatic impairment, hepatic cytolysis
24	F	6	6	Negative	At the age of 6 months - hypoglycemia, coma	Intermediate (precipitated by infection)	Hypotonia, disorders of consciousness, hypoglycemia, cardiomyopathy
25	M	6	6	Unknown	Unknown	Intermediate (precipitated by infection)	Hypoglycemia, cardiomyopathy, hypotonia
26	M	7	Unknown	Unknown	Unknown	Intermediate (precipitated by infection)	Unknown
27	M	7	Unknown	Negative	At the age of 4 months - occasional vomiting, cramps	Intermediate (precipitated by infection)	Hypotonia, hepatomegaly, hypoglycemia, hepatic cytolysis
28	M	7	7	Unknown	Unknown	Intermediate (precipitated by infection)	Hypoglycemia, hepatic cytolysis
29	M	7	Unknown	Unknown	Unknown	Intermediate (precipitated by infection)	Hepatomegaly, hepatic cytolysis

30	M	7	Unknown	Unknown	Unknown	Intermediate (precipitated by infection)	Hepatomegaly, hepatic cytolysis
31	F	7	7	Negative	At the age of 7 months - hypoglycemia	Intermediate (precipitated by infection)	Hypoglycemia, hepatic impairment (hepatomegaly, hepatic cytolysis), hypotonia
32	F	7	7	Negative	At the age of 7 months - hypoglycemia	Intermediate (precipitated by infection)	Hypoglycemia, hepatic impairment (hepatomegaly, hepatic cytolysis), hypotonia, cardiomyopathy
33	M	Retrospective diagnosis	0 (5 days)	Negative	At the age of 3 days - disorders of consciousness	Severe	Hypotonia, disorders of consciousness, cardiomyopathy
34	F	8	8	Positive (the sibling died of Reye's syndrome at the age of 6 months)	At the age of 6 months - increased levels of transaminases	Intermediate (precipitated by infection)	Hypotonia, hepatomegaly, hypoglycemia, cardiomyopathy, hepatic cytolysis
35	M	8	Alive	Negative	At the age of 8 months - hypoglycemia, hepatic cytolysis	Intermediate (precipitated by infection)	Hypoglycemia, hepatic impairment (hepatomegaly, hepatic cytolysis), hypotonia
36	F	8	8	Negative	At the age of 8 months - hypotonia, hepatomegaly, hepatic cytolysis	Intermediate (precipitated by infection)	Hypoglycemia, hepatic impairment (hepatomegaly, hepatic cytolysis), hypotonia, cardiomyopathy
37	M	8	Unknown	Unknown	Unknown	Intermediate (precipitated by infection)	Hypotonia, hypoglycemia, hepatomegaly
38	M	8	8	Positive	At the age of 7 months -	Intermediate (precipitated by	Hypotonia, hepatomegaly, hypoglycemia, cardiomyopathy, hepatic

					muscle weakness	infection)	cytolysis
39	F	8	Alive	Negative	At the age of 5 days - hypoglycemia. At the age of 7 months - hypoglycemia, hepatic cytolysis	Severe	Hepatomegaly, hepatic cytolysis, hypoproteinemia, hypoglycemia, anemia, hypotonia, cardiomyopathy
40	F	8	8	Negative	At the age of 9 months - hypoglycemia, hepatic cytolysis	Intermediate (precipitated by infection)	At the age of 8 months - hypoglycemia, hepatomegaly, hepatic cytolysis
41	F	9	Alive	Positive (the sibling died within the first month of life)	At the age of 9 months - hypoglycemia	Intermediate (precipitated by infection)	Hypoglycemia, hepatic impairment (hepatomegaly, hepatic cytolysis), hypotonia
42	M	9	Alive	Positive (the sibling died at the age of 8 months)	At the age of 9 months - hepatomegaly, hepatic cytolysis, hypotonia	Intermediate (precipitated by infection)	Hypoglycemia, hepatic impairment (hepatomegaly, hepatic cytolysis), anemia, hypotonia
43	M	9	Alive	Negative	At the age of 4 months - food refusal, hepatic cytolysis	Intermediate (precipitated by infection)	Hepatic cytolysis, anemia, motor impairment, hypotonia, duodenal atresia
44	M	9	Unknown	Unknown	Unknown	Intermediate (precipitated by infection)	Hypoglycemia, hepatic impairment (hepatomegaly, hepatic cytolysis), hypotonia
45	M	7	Alive	Negative	At the age of 7 months – hypoglycemia	Intermediate (precipitated by infection)	Hypoglycemia, hepatic impairment (hepatomegaly, hepatic cytolysis), hypotonia
46	F	10	13	Negative	At the age of 9 months - hepatic cytolysis,	Intermediate (precipitated by	Hypoglycemia, hepatic impairment (hepatomegaly, hepatic cytolysis),

					hepatomegaly, hypotonia	infection)	hypotonia, cardiomyopathy
47	F	10	Unknown	Unknown	Unknown	Intermediate (precipitated by infection)	Hepatic impairment, hypoglycemia
48	M	0 (4 days)	Alive	Positive (the sibling died within the first year of life)	No symptoms. The patient is receiving therapy since birth	Asymptomatic	No symptoms. The patient is receiving therapy
49	M	10	Alive	Negative	At the age of 8 months - hypoglycemia, hepatomegaly	Intermediate (precipitated by infection)	Hepatomegaly, hepatic cytolysis, hypoglycemia, metabolic acidosis,
50	M	11	Unknown	Unknown	Unknown	Intermediate (precipitated by infection)	Hypoglycemia, hepatic impairment (hepatomegaly, hepatic cytolysis), hypotonia
51	F	11	Alive	Negative	At the age of 10 months - disorders of consciousness, hypoglycemia	Intermediate (precipitated by infection)	Hypoglycemia, hepatic impairment (hepatomegaly, hepatic cytolysis), anemia, hypotonia
52	M	85	Unknown	Unknown	Muscle weakness	Mild	Chronic. Rhabdomyolysis, muscle weakness
53	M	166,1	Alive	Negative	At the age of 2 months - hypoglycemia	Mild	Below 15 years of age - two episodes of hypoglycemia and cardiomyopathy. At the age of 15 - metabolic crisis, hypoglycemia, rhabdomyolysis
54	M	318	Unknown	Unknown	Muscle weakness	Mild	Rhabdomyolysis, muscle weakness
